# The economic burden in terms of cost of illness and generic health-related quality of life of posttraumatic long bone non-unions among the adult population of the Netherlands from a societal perspective

**DOI:** 10.1007/s00068-026-03228-y

**Published:** 2026-06-10

**Authors:** L. C. A. van der Broeck, D. G. Shchurov, A. Gabrio, A. J. L. Lodewijks, M. Poeze, T. J. Blokhuis, S. Evers

**Affiliations:** 1 Department of Trauma Surgery, Maastricht University Medical Center, P. Debyelaan 25, Maastricht, 6229 HX The Netherlands; 2 Department of Surgery, NUTRIM School for Nutrition and Translational Research in Metabolism, Maastricht University Medical Center, Maastricht, The Netherlands; 3 Sechenov University, Moscow, Russia; 4 Department of Methodology and Statistics, FHML, Maastricht University, Peter Debeyeplein 1, Maastricht, 6229 HA The Netherlands; 5 Department of Health Services Research, Faculty of Health, Medicine and Life Sciences, CAPHRI, Maastricht University, Universiteitssingel 40, Maastricht, 6229 ER The Netherlands; 6 Trimbos Institute, Netherlands Institute of Mental Health and Addiction Utrecht, Utrecht, 3521 VS The Netherlands

**Keywords:** Cost of illness, Non-union, Societal perspective, Health-related quality of life, Posttraumatic

## Abstract

**Purpose:**

This study assessed the societal economic burden in terms of cost of illness and health-related quality of life (HRQoL) of posttraumatic long bone non-unions in the Netherlands.

**Methods:**

An incidence-based bottom-up approach was used, focusing on adult patients with posttraumatic long bone non-unions. The analysis included healthcare costs, patient and family costs, and productivity losses, measured using the iMCQ and iPCQ questionnaires. Cost evaluations followed Dutch costing guidelines, and productivity losses were calculated using the friction cost method. HRQoL was assessed with the EQ-5D-5L. A deterministic one-way sensitivity analysis varied baseline characteristics by ± 10%. Scenario analyses were conducted from healthcare and patient perspectives, as well as for patient subgroups.

**Results:**

Average costs per patient during the three months before the initial visit at a non-union clinic were €8,928 (healthcare, *N* = 78), €1,360 (patient and family, *N* = 58), and €4,313 (productivity losses, *N* = 59), the average of the observed data calculates to 10,831 (*N* = 44). The mean EQ-5D-5L utility score was 0.390 (± 0.29 SD). Subgroup analysis showed no significant cost increase for patients with infections or open fractures. However, a higher Non-Union Severity Score and a lower HRQoL were significantly associated with higher total costs.

**Conclusion:**

Posttraumatic long bone non-unions pose a substantial economic burden on Dutch society and have a tremendous impact on HRQoL. Severe non-unions and a lower quality of life were associated with increased costs, whereas initial fracture characteristics were not. These findings highlight the importance of effective preventive and therapeutic strategies to reduce the burden of posttraumatic long bone non-unions.

**Supplementary Information:**

The online version contains supplementary material available at 10.1007/s00068-026-03228-y.

## Introduction

Long bone fractures are associated with high morbidity worldwide and represent one of the most frequent reasons for hospitalization in trauma departments [1, 2]. One of the most impactful complications of fracture healing is the development of a non-union. A non-union is defined by the United States Food and Drug Administration as a fracture that fails to heal within nine months post-trauma, with no signs of progression within the last three months [3, 4]. Non-unions are particularly common in the lower extremities and are influenced by several risk factors, including smoking, infection, diabetes mellitus, and open fractures [5–9]. The estimated overall risk of developing a non-union is approximately 2–4% per fracture. However, certain risk factors, such as open fractures, are associated with a non-union risk of 20% [2, 5, 10]. These persistent healing failures can result in prolonged disability, repeated surgical interventions, and reduced health-related quality of life (HRQoL), placing a significant economic burden on healthcare systems and society [11–13].

Posttraumatic long bone non-unions have been shown to decrease the quality of life (QoL) by up to 43% versus the normative score for the general Dutch population, thereby resulting in a score even lower than in patients with end-stage colon cancer or lung cancer [14, 15]. Alongside the detrimentally low QoL, the treatment of non-unions requires significant healthcare resources, resulting in high direct costs for this condition [1]. However, the burden of non-unions extends beyond healthcare costs, also encompassing expenses for patients and their families, as well as productivity losses due to work absenteeism. These numbers have not yet been specified for posttraumatic long bone non-unions, but given the lengthy duration of this condition and the poor health-related quality of life (HRQoL), the additional costs are expected to be substantial.

In the current scientific literature, the healthcare costs associated with posttraumatic long bone non-unions were shown to be relatively high [1, 12, 16]. The total societal burden in terms of the cost of illness (CoI) and QoL is still unknown. Therefore, this study aims to assess the economic burden of posttraumatic long bone non-unions in the Netherlands from a societal perspective by evaluating the healthcare costs, patient and family costs, productivity costs, and the burden in terms of HRQoL.

## Methods

### Participants and data collection

This observational cohort study included all consecutive adult patients with a diagnosed posttraumatic long bone non-union, according to the FDA definition [3, 4]. Patients were recruited at the tertiary referral expert clinic for non-unions at the Maastricht University Medical Center (MUMC+), The Netherlands, from January 2020 to June 2024. Exclusion criteria included non-traumatic non-unions and the inability to speak or read the Dutch language. Patient characteristics and demographics were collected using the hospital registration data system. The Non-union Scoring system (NUSS) was used to classify the non-union severity at baseline. The NUSS uses three categories of parameters: the bone (e.g. bone defect, alignment, quality), the soft tissue (e.g. vascularization, skin defects) and overall patient status (e.g. diabetes, smoking, drugs). The NUSS score ranges from 0 (no complexity) to 100 (very high complexity) [17, 18]. At the expert non-union clinic intake, patients completed a series of validated questionnaires to assess quality of life and costs. These included the EQ-5D-5L [19], Medical Consumption Questionnaire (iMCQ) [20, 21], and Productivity Cost Questionnaire (iPCQ) [20]. These questionnaires adhere to a strict time horizon of the past three months at the time of application in order to decrease patient recall bias. This time horizon is applied over the entire assessment period.

**Fig. 1:**
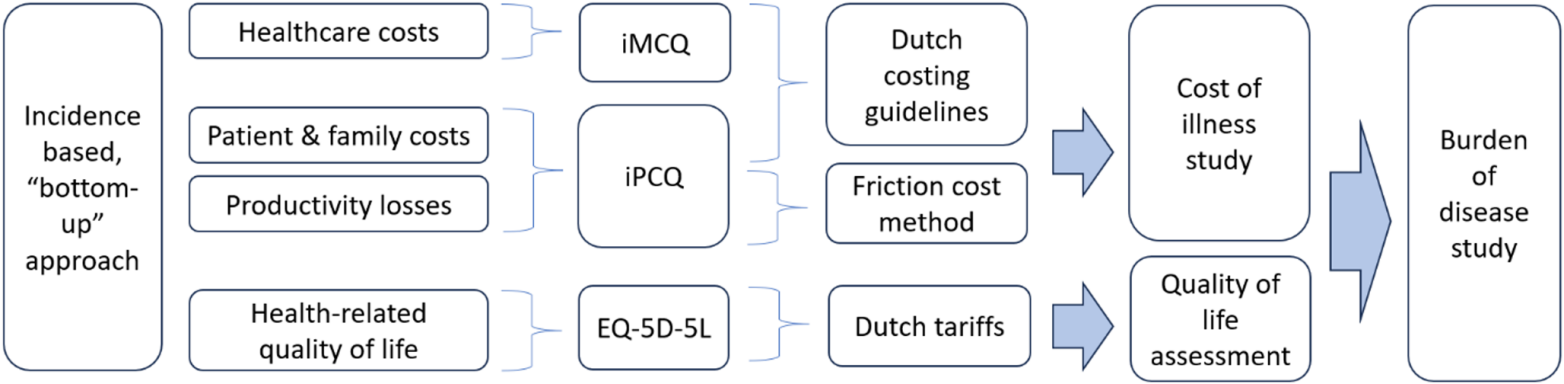
The overall conceptualization of this burden of disease study; Abbreviations: iMCQ - institute for Medical Technology Assessment Medical Consumption Questionnaire; iPCQ - institute for Medical Technology Assessment Productivity Cost Questionnaire; EQ-5D-5L - EuroQol 5 dimensions 5 levels questionnaire

A societal perspective was adopted for conducting both a Cost of Illness study (CoI) and a Quality of Life (QoL) assessment, as shown in Fig. 1. The CoI study evaluated costs three months before the initial visit, following a bottom-up incidence-based approach. Costs were identified, measured, valued, and summarized in compliance with Dutch healthcare economic guidelines and CHEERS 2022 standards [22–24]. Ethical approval was obtained from the local ethics committee, including informed consent from patients included in the analyses (METC22-3342).

The CoI analysis included healthcare costs (e.g., diagnostics, specialist visits, treatments, hospitalizations, and rehabilitation), patient and family costs (e.g., transportation and informal care), and productivity losses (e.g., absenteeism and presenteeism). Absenteeism was calculated as the number of workdays lost due to illness multiplied by the corresponding wage rate, whereas presenteeism was estimated as the reduction in productivity while at work (self-reported inefficiency) multiplied by the number of days worked and the wage rate. Healthcare service volumes were measured using the iMCQ, while the iPCQ was used to assess the patient and family costs and productivity losses [20]. Prices were based on the 2023 Dutch costing guidelines, adjusted for inflation using the consumer price index, while medications were valued using the lowest prices per unit, derived from the Dutch national institute Zorginstituut Nederland [25]. Informal care costs were based on duration and hourly rates for informal caregiving, while productivity losses were valued using the friction cost method with the 2023 labor cost index [26, 27]. The valuation of drug therapy was based on the lowest prices per unit of medication following the Dutch guidelines [26]. Prior surgical treatment costs were assessed based on the publicly available tariffs for medical specialty care provided by the Dutch Healthcare Authority (Nederlandse Zorgautoriteit). A breakdown of the type of service, unit price, and evidence source used to calculate costs is shown in Supplementary Table 1. No discounting was applied due to the less-than-one-year time horizon of the study. HRQoL was measured using the Dutch version of the EQ-5D-5L questionnaire. The EQ-5D-5L Utility scores were measured using the Dutch value set [28, 29].

### Statistical analysis

Patients were excluded from the analysis if a patient did not complete all three questionnaires; their answers were considered as missing in the base-case analysis. Statistical analysis was carried out using R Statistical Software (v4.2.2). Patient characteristics were summarized in terms of mean (standard deviation) for continuous variables, and frequencies for each level of the categorical variables. The primary analysis was conducted based on the observed data alone (base-case analysis) and results were reported in terms of: (1) descriptive statistics for key cost and health variables; (2) linear regression coefficients capturing the association between different types of costs and the following variables: infection status (yes/no), NUSS, open fracture status (yes/no), EQ-5D-5L utility score. Coefficients (and 95%CI) estimated from each regression are obtained after controlling for potential confounders. All analyses used linear regression models starting from a predefined full model including clinically relevant predictors (“Infection_status”, “Gender”, “Age”, “Diabetes”, “Smoking”, “Daily_living”, and “Paid_work”), selected based on literature, expert opinion, and descriptive analyses. A backward selection procedure guided by AIC/BIC was applied to obtain parsimonious, outcome-specific final models. A secondary analysis was conducted to assess the sensitivity of the results to missingness uncertainty via multiple imputation by chained equation (MICE) [30] with a predictive mean matching method and a total of 25 imputations under a missing at random assumption (MAR) [31]. Combined results across imputed datasets were obtained via Rubin’s rules and are reported in terms of mean or linear regression coefficient estimates (and 95% CI). Regression results from the secondary analysis were produced using the same model specification as in the primary analysis, with the only difference that comparisons in terms of open fracture status are also adjusted for the type of open fracture experienced (omitted in the primary analysis due to many missing values). For all regression analyses, statistical results are reported in terms of coefficient estimates, 95%CI, and p-values assuming a significance level of 5%. Due to the explorative nature of the study, no adjustment for multiple comparisons is made.

## Results

### Patient characteristics

During the study period, a total of 133 patients with non-unions were assessed at the clinic. Of these, seventy-eight were included in the analysis. Thirty-eight patients were excluded because they did not complete all three questionnaires. An additional seventeen patients were not included because of non-union sites other than long bones or because the cause of the fractures was not posttraumatic. The patient population was predominantly male (64%), and the mean age was 53 years (SD 15.5). More than half of the patients (57%) were working as self-employed or employees, 23% were unemployed or disabled, and nearly 20% were on retirement. Among disabled patients, 50% reported that they were completely disabled, and the rest reported that they were partially disabled. Key patient characteristics are summarized in Table 1.

**Table 1:** Key characteristics of the study patients with posttraumatic long bone non-unions in the Netherlands, 2020-2024

Patient Characteristics	*n*	Frequency (Percent) or mean (SD)
Age	78	53.4 (± 15.5)
Gender,	78	
Male		50 (64%)
Female		28 (36%)
Diabetes, yes	78	5 (6.6%)
Smoking, yes	76	16 (21%)
Infection, yes	62	34 (55%)
Open fracture, yes	73	24 (33%)
Gustillo-Anderson grade 1	24	8 (33%)
Gustillo-Anderson grade 2–3 A	24	9 (37.5%)
Gustillo-Anderson grade 3B	24	4 (17%)
Unkown	24	3 (12.5%)
Having paid work, yes	76	43 (57%)
Main status in daily living	76	
Employee		32 (42%)
Self-employed		10 (13%)
Unemployed		5 (7%)
Lifelong disabled		15 (20%)
Retirement		14 (18%)

### Cost of illness

The total healthcare costs per patient were calculated to be €8,928.17 (SD, 16,459.07) for the three months preceding admission to the clinic. Major contributors to these costs were previous surgical interventions, admission to a rehabilitation center, hospital admission, and rehabilitation treatment consultations, as shown in Table 2.

**Table 2:** The costs, per subcategory, associated with posttraumatic long bone non-unions per patient for three months, based on the observed data

Cost component	Mean costs (± SD), €	Median (IQR)
*Healthcare costs*		
Diagnostics* (*N* = 78)	82.17 (± 0.00)	
Outpatient visits 1st line (*N* = 78)	798.24 (± 738.00)	
Outpatient visits hospital (*N* = 78)	324.18 (± 335.52)	
Home care (*N* = 78)	492.37 (± 1,729.91)	
Medication (*N* = 78)	48.53 (± 72.13)	
Ambulance (*N* = 78)	26.23 (± 132.00)	
Emergency room visit (*N* = 78)	44.63 (± 132.44)	
Care in a hospital, overnight (*N* = 78)	1,259.81 (± 3,878.38)	
Care in a psychiatric institution (*N* = 78)	182.77 (± 1,614.18)	
Care in a rehabilitation center (*N* = 78)	1,310.59 (± 9,315.28)	
Surgical treatment last 3 months (*N* = 11)	3,518.21 (± 10,377.89)	
PSOS	1,064.33 (± 6,603.46)	
Dynamisation	696.73 (± 3,506.20)	
Debridement procedure	237.63 (± 2,098.68)	
External fixator	308.08 (± 1,550.35)	
Removal of osteosynthetic	928.97 (± 4,021.53)	
Soft tissue procedure	282.46 (± 2,494.63)	
**Healthcare costs**,** total (*N* = 78)**	**8**,**928.17 (± 16**,**459.07)**	**1**,**520.65 (4**,**386.50)**
*Patient and family costs*		
Informal care (*N* = 61)	1,203.44 (± 2,407.92)	
Transportation (*N* = 72)	126.12 (± 132.75)	
**Patient and family costs**,** total (*N* = 58)**	**1**,**360.48 (± 2**,**460.98)**	**1**,**294.61 (4**,**010.43)**
*Productivity losses*		
Absenteeism (*N* = 73)	6,255.19 (± 9,578.29)	
Presenteeism (*N* = 59)	444.12 (± 1,294.90)	
**Productivity losses**,** total (*N* = 59)**	**4**,**313.36 (± 9**,**296.11)**	
**Grand Total Cost (** *N* ** = 44)**	**10**,**831.33 (± 17**,**972.46)**	**2**,**544.91 (7**,**360.51)**

The average duration of informal care was estimated at 61.7 h per patient during the four weeks prior to the first visit in the non-union clinic, leading to an average informal care cost of €1,203. The average cost of transportation to and from healthcare organizations was estimated to be €126 for three months per adult patient with posttraumatic long bone non-union.

In the four weeks prior to completing the questionnaire, patients were estimated to have missed an average of 21 working days (151 h). About 63% of the patients worked while experiencing discomfort from the condition, with an associated reduction in productivity of approximately 60%. Considering the mean productivity costs per hour - €41.40 [26], the average cost related to absenteeism and presenteeism was estimated to be €6,255 and €444 per patient, respectively.

The overall mean cost of the posttraumatic long bone non-unions was calculated to be €10,831.33 (SD 17,972.46) per patient (*N* = 44) over three months before admission to the non-union clinic. The cost categories that contributed the most to the total cost of the disease were productivity losses and healthcare costs. The frequencies of the use of healthcare services and informal care, as well as the number of hours of absenteeism and presenteeism per patient, are shown in the Supplementary Data. Results related to healthcare costs, patient and family costs, productivity losses, and the overall costs associated with posttraumatic long bone non-unions are summarized and presented in Table 2.

### Health-related quality of life

**Fig. 2:**
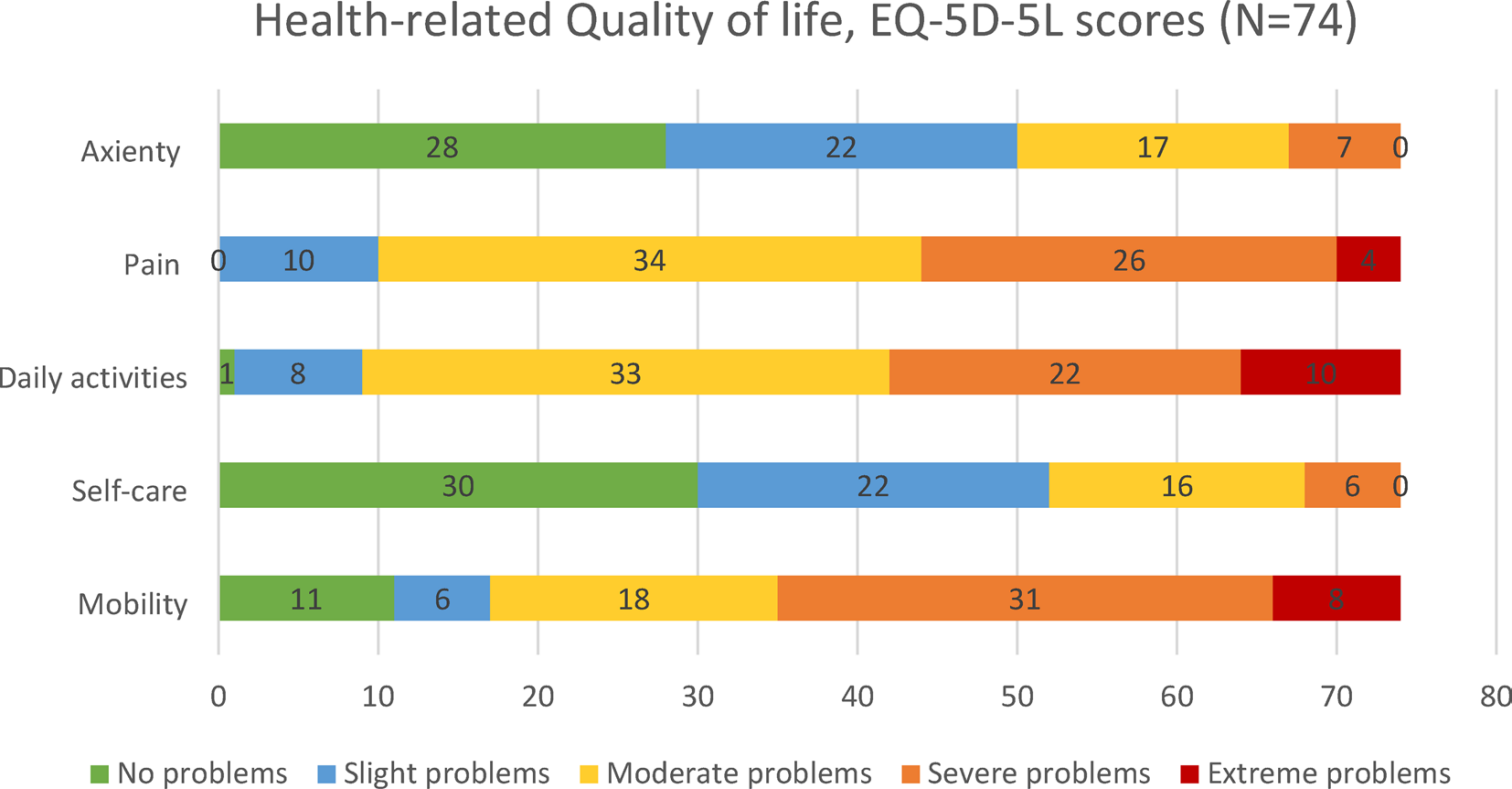
The EQ-5D-5L utility score frequencies among patients with non-union, based on the observed data. Data is depicted by each EQ-5D-5L domain

The mean EQ-5D-5L utility index scores across all included patients were 0.390 (SD 0.29). This was mainly driven by low scores associated with a lack of mobility, the inability to perform daily activities, and the presence of pain. Interestingly, overall anxiety levels remained relatively low in this patient population. The scores per dimension are shown in Fig. 2.

### Sensitivity analyses

Following multiple imputation (primary analysis in supplemental Table 7), a secondary analysis was conducted using a series of univariate linear regression models to examine cost differences across specific subgroups. Key variables of interest included infection status (yes/no), presence of an open fracture (yes/no), performance of the NUSS procedure, and HRQoL. No statistically significant associations were observed between total costs and either infection (95% CI -13103 — 7127) or open fracture (CI 95% -9723 — 9625) subgroups. In contrast, lower HRQoL scores were significantly associated with higher total costs (95% CI -39775 — -5957). Although the NUSS score showed a significant influence on several individual cost categories, particularly previous surgical treatment (95% CI 23 — 86), it was not significantly associated with the overall total costs (95% CI -41 — 694), as shown in Table 3. See Supplementary Data Tables 3, 4, 5 and 6 for a full breakdown of the results from the sensitivity analyses. The primary analysis, a series of univariate linear regression analyses fitted to the observed data (after removal of all missing cases) for different outcomes (cost and EQ-5D-5L) to assess their association with the variables infection, open fracture, NUSS, and HRQoL, showed similar results. Data available by author request.

**Table 3:** Summary estimates (mean difference/regression coefficient), p-value, 95% confidence intervals and baseline variables included in the regression analysis assessing the association between total cost and infection (yes/no), open fracture (no/yes), NUSS and EQ-5D-5L index, based on the imputed data

Outcome	Total cost difference	*P*-value	95% CI (Lower)	95% CI (Upper)
Infection (yes/no)	-9046.50 (mean difference)	0.557	-13,103	7128
Open fracture (yes/no)	49.27 (mean difference)	0.992	-9723	9625
NUSS	326.52 (regression coefficient)	0.081	-41	694
EQ-5D-5L index	-22866.52 (regression coefficient)	0.009	-39,776	-5957

## Discussion

This study quantified the economic burden of posttraumatic long bone non-unions in the Netherlands in terms of CoI and generic HRQoL from a societal perspective. The overall cost of illness was calculated to be €10,831.33 per patient for three months prior to their active treatment in our tertiary referral hospital, thus representing the societal footprint of having a non-union yet before final treatment has been initiated. The healthcare costs and productivity losses were generally identified as the main total cost contributors, with productivity losses (absenteeism €6,255.19 (SD ± €9,578.29)) being associated with the highest costs. In terms of HRQoL, the calculated average EQ-5D-5L utility score of the study population was 0.390 (SD 0.29) and was mainly driven by lack of mobility, pain, and inability to execute daily activities.

The sensitivity analysis revealed no substantial influence on the overall cost in the infected non-union group or in non-unions that resulted from an initial open fracture. However, having a more severe non-union has been shown to significantly increase the prior treatment cost by €54.50 per point on the NUSS scale. Unfortunately, the primary analysis was not able to demonstrate this significant increase. Moreover, a lower quality of life score was revealed to increase the cost by €228.66 per 0.1 decrease in utility score. Notably, the majority of this cost increase was attributed to the productivity losses (€-109.60 (*p* = 0.007) in secondary analysis; €-123.23 (*p* = 0.005) in primary analysis). This data highlights the well-known association between active engagement in society and health-related quality of life (HRQoL), as described by Bouwmans et al. [32]. Lower health-related quality of life (HRQoL) was associated with higher healthcare costs. However, given the cross-sectional and observational nature of this study, the direction of this relationship cannot be determined, and reverse causality or confounding by disease severity is likely.

The healthcare costs associated with posttraumatic long bone non-unions within a time horizon of 3 months, without initiated final active treatment in our hospital, were nearly equal to the healthcare costs that the overall Dutch healthcare system spends per inhabitant during a year, which is estimated to be €5,600 [33], emphasizing the burden of this condition even further. Nevertheless, substantial variability exists within this patient sample. The data exhibit a high standard deviation and a wide range in healthcare costs (range: €82.17 to €80,409.37), indicating the presence of outlier cases with exceptionally high expenditures. However, such skewness is inherent to healthcare cost data, and the mean remains the most appropriate summary measure as it captures the full economic burden at the population level, despite being influenced by high-cost cases. Overall, the observed variability is not unexpected in this context but rather reflects the clinical heterogeneity and complexity of the patient population in this study. Furthermore, the results are primarily based on observed data, with a considerable amount of missing values. As a result, complete-case analysis was required for the calculation of total costs, limiting the grand total to *N* = 44. This explains why the final aggregated costs are based on a smaller subset than the observed subgroup results (*N* = 78;58;59). While this approach is methodologically necessary to ensure valid summation across all cost categories, it reduces the effective sample size and may decrease the precision of the estimates. Consequently, the reported total costs are likely conservative and may represent an underestimation of the true economic burden in this patient population. Moreover, when considering the minimally clinically important difference in utility score (0.03 units [34]), the QoL of this patient population is drastically lower than that of the general adult population in the Netherlands (0.869) [29, 34], even lower than the utility score reported by Vincken et al. in 2023, of a similar patient population, 0.490 (± 0.261 SD) [14].

Since this study is historically the first estimation of the costs associated with posttraumatic long bone non-unions in the Netherlands, a direct comparison of results from other studies is difficult. However, previously published studies performed in other countries showed that productivity losses account for approximately 55% of the total costs associated with an uneventful tibial fracture and 80% of the total costs of injury [35, 36]. An overview of the financial impact of non-unions was reported by Hak et al., summarizing the average healthcare costs of the treatment of an established long bone non-union at CN$11,800, US$11,333, and £29,204 [8]. Furthermore, few studies have incorporated HRQoL data into CoI assessments, making our inclusion of utility scores a notable contribution. The severely reduced HRQoL in our cohort is consistent with findings from other European studies, which often report EQ-5D utility scores below 0.5 in patients with tibial or femoral non-unions [37, 38]. However, the average utility score of 0.390 found here is among the lowest reported, underscoring the profound functional and psychosocial impact of this condition. Furthermore, unlike most health economics studies that use modeling, this study was designed to be prospective, bottom-up, and grounded in real-world data. The relatively large cohort of posttraumatic long bone non-unions is another strength of this study, providing relatively rigid data on this specific condition. This study focuses on the Dutch cost-of-illness of post-traumatic long bone non-unions, due to a renowned limitation in European economic evaluations, where heterogeneity in cost-estimation methodologies and lack of standardization hinder valid cross-country comparisons; nevertheless, this standardized Dutch evaluation provides valuable insights that are relevant and informative for the broader European context. Despite these constraints, the findings clearly demonstrate that post-traumatic non-unions impose substantial costs and a significant societal burden, underscoring the need for increased awareness and stronger emphasis on prevention across Europe.

The use of a 3-month recall period, in line with the validated iMCQ/iPCQ methodology, may underestimate the total societal burden of post-traumatic non-unions, as patients often experience a prolonged and resource-intensive disease course extending beyond this timeframe. However, this approach was chosen to minimize recall bias and ensure reliable patient-reported data, thereby providing a standardized and methodologically robust estimate of healthcare utilization in a clinically relevant pre-referral period. Moreover, the patient population in this study represents a complex case series, due to the nature of our referral centre, as is illustrated by a high mean NUSS score. Although this may raise concerns regarding representativeness, we mitigated potential overestimation by retrospectively capturing costs prior to referral, thereby reflecting the full treatment pathway rather than only tertiary-level interventions. Overestimation would be more likely if costs were primarily driven by advanced reconstructive procedures such as induced membrane techniques with bioactive materials, however, these were not the basis of the pre-referral cost accumulation. Nevertheless, within this heterogeneous patient sample, our findings may still represent an overestimation for less complex cases.

Furthermore, while the use of Dutch costing guidelines and the friction cost method may limit direct transferability to other healthcare systems, these standardized methodologies enhance internal validity and comparability with other economic evaluations. Although absolute cost estimates may differ between countries, the overall conclusion is expected to be consistent across comparable high-income healthcare settings. 

Despite these limitations, the data presented is crucial for increasing awareness of the burden of post-traumatic non-unions to the healthcare systems, society, and the patients themselves. This substantial impact on the economy and on our patients’ well-being emphasizes the need for adequate prevention, a multimodal treatment plan, and proper reimbursement for post-traumatic long bone non-unions in the Netherlands.

## Supplementary Information

Below is the link to the electronic supplementary material.

Supplementary Material 1

Supplementary Material 2

Supplementary Material 3

Supplementary Material 4

Supplementary Material 5

Supplementary Material 6

Supplementary Material 7

## Data Availability

The data supporting the findings of this study were generated by the authors at a local hospital, as described in the manuscript. Due to patient privacy considerations and institutional regulations, the data are not publicly available. De-identified data may be made available from the corresponding author upon reasonable request and with permission from the relevant institutional authority.
