# Supplementary material for: The economic burden in terms of cost of illness and generic health-related quality of life of posttraumatic long bone non-unions among the adult population of the Netherlands from a societal perspective
Source: Eur J Trauma Emerg Surg. 2026 Jun 10;52(1):183. doi: 10.1007/s00068-026-03228-y (PMC13253652; doi:10.1007/s00068-026-03228-y)
Supplement: Supplementary file 1 — Supplementary Material 1 [file 68_2026_3228_MOESM1_ESM.docx]

**Supplementary Table 1.** The input data used for the calculation of the costs in the research

| Indicator name | Value | Source of data |
| --- | --- | --- |
| Diagnostics | | |
| X-ray, per one scan | €85.29 | [26] |
| Medical, paramedical and mental care in outpatient settings | | |
| GP visit, average, per consultation | €44.96 | [26] |
| Appointment with a company physician, per consultation | €44.96 | Assumed to be equal to a GP visit |
| Physiotherapy per session | €40.37 | [26] |
| Occupational therapy, per session | €25.24 | [26] |
| Homeopath advice, per consultation | €40.37 | Assumed to be equal to a physiotherapy per session |
| Dietary advice, per session (consultation) | €25.64 | [26] |
| Psychologist advice, per consultation | €102.36 | [26] |
| Social worker visit, per one appointment | €131.83 | [26] |
| Outpatient visit to medical specialists, per consultation | €124.56 | [26] |
| The fee for issuing a medicine to a patient, per issue | €6.50 | [26] |
| Ambulance and inpatient care | | |
| Emergency transport, per one transportation | €681.97 | [26] |
| Emergency room, per visit | €267.80 | [26] |
| Care in a hospital, per day | €668.47 | [26] |
| Care in a psychiatric institution, per day | €339.43 | [26] |
| Rehabilitation | | |
| Rehabilitation treatment consultation, per one consultation | €360.19 | [26] |
| Care in a rehabilitation center, day | €881.26 | [26] |
| Home care | | |
| Household help, per hour | €34.00 | [26] |
| Personal care, per hour | €59.77 | [26] |
| Nursing, per hour | €77.85 | [26] |
| Informal care | | |
| Informal care, per hour | €19.51 | [26] |
| Patient’s transportation | | |
| Distance from home to an outpatient clinic with medical specialists and psychologists | 4.8 km | [26], assumption |
| Distance from home to a hospital or rehabilitation center | 7.1 km | [26], assumption |
| Distance from home to a GP practice clinic | 1.0 km | [26] |
| Distance from home to physiotherapist, occupational therapist, dietitian, homeopath, social worker or company physician | 2.2 km | [26], assumption |
| Car, price per kilometer | €0.27 | [26] |
| Car, parking fee per visit | €4.07 | [26] |
| Cost of time spent by informal carers, replacement costs per hour | €18.68 | [26] |
| Productivity losses | | |
| Productivity costs per hour | €41.40 | [26] |
