# Supplementary material for: The economic burden in terms of cost of illness and generic health-related quality of life of posttraumatic long bone non-unions among the adult population of the Netherlands from a societal perspective
Source: Eur J Trauma Emerg Surg. 2026 Jun 10;52(1):183. doi: 10.1007/s00068-026-03228-y (PMC13253652; doi:10.1007/s00068-026-03228-y)
Supplement: Supplementary file 2 — Supplementary Material 2 [file 68_2026_3228_MOESM2_ESM.docx]

**Supplementary Table 2.** The use of healthcare and informal care services, and hours of productivity loss, per patient

| Resource utilization, units | Frequency | Range (min-max) |
| --- | --- | --- |
| *Healthcare services* | | |
| Diagnostics | | |
| X-ray, examinations | 1* | 1-1 |
| Medical, paramedical and mental care in outpatient settings | | |
| GP, visits | 2.19 | 0-10 |
| Company physician, visits | 0.64 | 0-4 |
| Physiotherapy, sessions | 11.75 | 0-48 |
| Occupational therapy, sessions | 0.78 | 0-20 |
| Homeopath advice, consultations | 0.16 | 0-5 |
| Dietary advice, consultations | 0.18 | 0-9 |
| Psychologist advice, consultations | 0.7 | 0-14 |
| Social worker, visits | 0.79 | 0-15 |
| Medical specialists, visits | 2.64 | 0-14 |
| Ambulance and inpatient care (without rehabilitation) | | |
| Ambulance, number of transportations | 0.04 | 0-1 |
| Emergency room, visits | 0.17 | 0-3 |
| Care in a hospital (overnight), days | 1.96 | 0-28 |
| Care in a psychiatric institution (overnight), days | 0.56 | 0-42 |
| Rehabilitation | | |
| Rehabilitation treatment, consultations | 2.39 | 0-65 |
| Care in a rehabilitation center (overnight), days | 1.55 | 0-90 |
| Home care | | |
| Household help, hours | 4.05 | 0-58.5 |
| Personal care, hours | 0.18 | 0-9.75 |
| Nursing, hours | 4.5 | 0-180 |
| *Patient and family resources* | | |
| Informal care, hours | 61.68 | 0-784 |
| Transportation (excluding ambulance) | See the frequencies of healthcare services | |
| *Loss of productivity* |  |  |
| Absenteeism, hours | 151.09 | 0-1028.57 |
| Presenteeism, hours | 10.73 | 0-205.71 |
