# Supplementary material for: The economic burden in terms of cost of illness and generic health-related quality of life of posttraumatic long bone non-unions among the adult population of the Netherlands from a societal perspective
Source: Eur J Trauma Emerg Surg. 2026 Jun 10;52(1):183. doi: 10.1007/s00068-026-03228-y (PMC13253652; doi:10.1007/s00068-026-03228-y)
Supplement: Supplementary file 3 — Supplementary Material 3 [file 68_2026_3228_MOESM3_ESM.docx]

**Supplementary Table 3.** Summary estimates (mean difference), p-value, 95 % confidence intervals and baseline variables included in the regression analysis assessing the association between different outcomes (first column) and infection status (yes/no) based on the imputed data.

|  | Mean difference | p-value | 95% CI (lower) | 95% CI (upper) | Adjusted variables |
| --- | --- | --- | --- | --- | --- |
| Outpatient | -193.707 | 0.288 | -555.41 | 167.995 | Age |
| Homecare | 270.53 | 0.5 | -525.927 | 1066.988 | Smoking |
| Surgtreat | -974.536 | 0.692 | -5863.511 | 3914.439 | - |
| Other | -1414.687 | 0.552 | -6147.307 | 3317.934 | Diabetes |
| prodloss | -270.664 | 0.899 | -4509.354 | 3968.026 | Paid_work |
| travelexp | -31.545 | 0.397 | -105.858 | 42.768 | Paid_work |
| informalcare | -187.756 | 0.807 | -1731.895 | 1356.382 | Gender, Smoking |
| total hc | -2082.097 | 0.588 | -9718.459 | 5554.266 | Diabetes |
| total f&p | -95.286 | 0.898 | -1585.855 | 1395.282 | Paid_work |
| total | -2987.911 | 0.557 | -13103.471 | 7127.65 | Paid_work |
| eq5d | 0.038 | 0.612 | -0.111 | 0.186 | Paid_work |
