# Supplementary material for: The economic burden in terms of cost of illness and generic health-related quality of life of posttraumatic long bone non-unions among the adult population of the Netherlands from a societal perspective
Source: Eur J Trauma Emerg Surg. 2026 Jun 10;52(1):183. doi: 10.1007/s00068-026-03228-y (PMC13253652; doi:10.1007/s00068-026-03228-y)
Supplement: Supplementary file 4 — Supplementary Material 4 [file 68_2026_3228_MOESM4_ESM.docx]

**Supplementary Table 4.** Summary estimates (mean difference), p-value, 95 % confidence intervals and baseline variables included in the regression analysis assessing the association between different outcomes (first column) and open fracture (no/yes) based on the imputed data.

|  | Mean difference | p-value | 95% CI (lower) | 95% CI (upper) | Adjusted variables |
| --- | --- | --- | --- | --- | --- |
| Outpatient | 266.105 | 0.143 | -92.578 | 624.788 | - |
| Homecare | 369.379 | 0.435 | -571.713 | 1310.472 | Gender, Diabetes, Daily_living, Paid_work |
| Surgtreat | -1334.644 | 0.597 | -6349.68 | 3680.392 | Gender, Smoking |
| Other | -1143.829 | 0.624 | -5777.315 | 3489.658 | Diabetes |
| prodloss | 2152.479 | 0.295 | -1920.156 | 6225.113 | Paid_work |
| travelexp | 48.116 | 0.147 | -17.386 | 113.617 | Paid_work |
| informalcare | -186.127 | 0.797 | -1633.146 | 1260.893 | - |
| total hc | -2016.515 | 0.596 | -9578.738 | 5545.708 | Gender, Diabetes, Smoking |
| total f&p | -111.849 | 0.877 | -1554.537 | 1330.84 | Paid_work |
| total | -49.272 | 0.992 | -9723.801 | 9625.257 | Paid_work |
| eq5d | -0.088 | 0.23 | -0.234 | 0.058 | Paid_work |
