# Supplementary material for: The economic burden in terms of cost of illness and generic health-related quality of life of posttraumatic long bone non-unions among the adult population of the Netherlands from a societal perspective
Source: Eur J Trauma Emerg Surg. 2026 Jun 10;52(1):183. doi: 10.1007/s00068-026-03228-y (PMC13253652; doi:10.1007/s00068-026-03228-y)
Supplement: Supplementary file 5 — Supplementary Material 5 [file 68_2026_3228_MOESM5_ESM.docx]

**Supplementary Table 5.** Summary estimates (regression coefficient), p-value, 95 % confidence intervals and baseline variables included in the regression analysis assessing the association between different outcomes (first column) and NUSS based on the imputed data.

|  | Regression coefficient | p-value | 95% CI (lower) | 95% CI (upper) | Adjusted variables |
| --- | --- | --- | --- | --- | --- |
| Outpatient | -0.436 | 0.95 | -14.159 | 13.287 | - |
| Homecare | 58.008 | 0 | 29.823 | 86.193 | - |
| Surgtreat | 54.473 | 0.001 | 22.97 | 85.975 | Gender, Smoking |
| Other | 45.814 | 0.607 | -131.152 | 222.779 | Diabetes |
| prodloss | 92.902 | 0.256 | -69.24 | 255.043 | Paid_work |
| travelexp | 0.945 | 0.473 | -1.671 | 3.56 | Paid_work |
| informalcare | 28.508 | 0.342 | -31.611 | 88.628 | - |
| total hc | 225.454 | 0.146 | -80.727 | 531.635 | Gender, Diabetes, Smoking |
| total f&p | 28.865 | 0.335 | -30.995 | 88.725 | Paid_work |
| total | 326.519 | 0.081 | -40.799 | 693.836 | Paid_work |
| eq5d | -0.004 | 0.117 | -0.01 | 0.001 | Paid_work |
