# Supplementary material for: The economic burden in terms of cost of illness and generic health-related quality of life of posttraumatic long bone non-unions among the adult population of the Netherlands from a societal perspective
Source: Eur J Trauma Emerg Surg. 2026 Jun 10;52(1):183. doi: 10.1007/s00068-026-03228-y (PMC13253652; doi:10.1007/s00068-026-03228-y)
Supplement: Supplementary file 6 — Supplementary Material 6 [file 68_2026_3228_MOESM6_ESM.docx]

**Supplementary Table 6.** Summary estimates (regression coefficient), p-value, 95 % confidence intervals and baseline variables included in the regression analysis assessing the association between different outcomes (first column) and EQ5D based on the imputed data.

|  | Regression coefficient | p-value | 95% CI (lower) | 95% CI (upper) | Adjusted variables |
| --- | --- | --- | --- | --- | --- |
| Outpatient | -639.437 | 0.043 | -1257.05 | -21.825 | Daily_living |
| Homecare | -1146.357 | 0.197 | -2912.943 | 620.23 | Gender, Diabetes, Paid_work |
| Surgtreat | -4597.247 | 0.33 | -13971.508 | 4777.015 | - |
| Other | -5955.968 | 0.128 | -13678.565 | 1766.63 | Diabetes |
| prodloss | -10961.657 | 0.007 | -18737.901 | -3185.413 | Gender, Age, Diabetes, Smoking, Daily_living, Paid_work |
| travelexp | -29.103 | 0.624 | -147.052 | 88.847 | - |
| informalcare | -2899.523 | 0.023 | -5371.903 | -427.143 | - |
| total hc | -12294.683 | 0.08 | -26129.535 | 1540.168 | Diabetes |
| total f&p | -2854.978 | 0.027 | -5375.428 | -334.528 | Paid_work |
| total | -22866.525 | 0.009 | -39775.575 | -5957.476 | Paid_work |
