# Supplementary material for: The economic burden in terms of cost of illness and generic health-related quality of life of posttraumatic long bone non-unions among the adult population of the Netherlands from a societal perspective
Source: Eur J Trauma Emerg Surg. 2026 Jun 10;52(1):183. doi: 10.1007/s00068-026-03228-y (PMC13253652; doi:10.1007/s00068-026-03228-y)
Supplement: Supplementary file 7 — Supplementary Material 7 [file 68_2026_3228_MOESM7_ESM.docx]

**Supplementary data**

**Table 7:** Summary estimates (proportions/means) and 95 % confidence intervals based on the pooled data after implementation of MI. For all binary variables, proportions are reported for category 1=yes; for all categorical variables, a series of dummy variables asosciated with each category are created and summarised in terms of their proportions; for all continuous variables, average estimates are reported.

|  | **Estimate** | **95% CI (lower)** | **95% CI (upper)** |
| --- | --- | --- | --- |
| Diabetes | 0.07 | 0.03 | 0.16 |
| Infection_status | 0.52 | 0.40 | 0.64 |
| Smoking | 0.22 | 0.14 | 0.33 |
| Daily_living_emp | 0.40 | 0.30 | 0.51 |
| Daily_living_entr | 0.12 | 0.06 | 0.21 |
| Daily_living_unemp | 0.06 | 0.03 | 0.15 |
| Daily_living_disab | 0.20 | 0.12 | 0.30 |
| Daily_living_retir | 0.19 | 0.12 | 0.30 |
| Daily_living_other | 0.03 | 0.01 | 0.10 |
| Typefract_23a | 0.21 | 0.12 | 0.35 |
| Typefract_3bc | 0.41 | 0.29 | 0.54 |
| Typefract_1 | 0.38 | 0.26 | 0.51 |
| Paid_work | 0.56 | 0.45 | 0.67 |
| openfracture | 0.34 | 0.24 | 0.45 |
| NUSS | 41.04 | 38.02 | 44.06 |
| N_hours_worked_day | 4.05 | 3.16 | 4.93 |
| N_work_days_absent | 21.52 | 14.50 | 28.54 |
| Presenteeism_hours | 13.09 | 4.85 | 21.33 |
| Absenteeism_hours | 151.99 | 98.68 | 205.30 |
| N_days_unpaid_work | 12.52 | 9.43 | 15.61 |
| N_hours_helped_by_others | 3.73 | 2.44 | 5.02 |
| Informal_care_hours | 66.19 | 33.69 | 98.69 |
| N_visits_specialists_and_psychologist | 3.36 | 2.46 | 4.26 |
| N_visits_physio_ergo_diet_homeo_social_comp | 14.24 | 11.05 | 17.44 |
| N_hosp_rehab_or_psychiatric | 3.30 | 0.89 | 5.70 |
| N_app_with_GP | 2.17 | 1.59 | 2.74 |
| Total_healthcare_costs | 8928.17 | 5215.70 | 12640.63 |
| Cost_absenteeism | 6292.31 | 4085.29 | 8499.32 |
| Cost_presenteeism | 541.93 | 200.68 | 883.19 |
| Travel_expenses_GP | 9.99 | 7.34 | 12.64 |
| Travel_exp_hosp_or_rehab | 26.06 | 7.06 | 45.07 |
| Trav_exp_physio_ergo_diet_homeo_soc_comp | 74.89 | 58.08 | 91.70 |
| Travel_expenses_speci_psycholog | 22.36 | 16.36 | 28.37 |
| Cost_informal_care | 1291.33 | 657.20 | 1925.47 |
| Total_productivity_losses | 6834.24 | 4483.98 | 9184.50 |
| Total_travel_expenses | 133.31 | 102.26 | 164.35 |
| Total_patient_and_family_costs | 8258.88 | 5799.09 | 10718.66 |
| TOTAL_COSTS | 17187.04 | 12503.01 | 21871.07 |
| EQ5D | 0.39 | 0.32 | 0.45 |

**Figure 3: Missingness patterns in key partially-observed variables in the data set.**

The table below shows all possible patterns of missing counts among the same variables as well as the overall proportions of missingness by pattern.

Combinations Count Percent

11 1:0:0:0:0:0:0:0:0:0:0:0:0 19 24.358974

14 1:0:0:0:1:0:0:0:0:0:0:0:0 13 16.666667

17 1:0:0:0:1:1:0:0:0:0:0:0:0 8 10.256410

4 0:0:0:0:1:0:0:0:0:0:0:0:0 5 6.410256

7 0:0:0:0:1:1:0:0:0:0:0:0:0 4 5.128205

1 0:0:0:0:0:0:0:0:0:0:0:0:0 4 5.128205

16 1:0:0:0:1:0:0:0:1:1:1:1:0 3 3.846154

15 1:0:0:0:1:0:0:0:0:1:1:1:0 3 3.846154

23 1:1:0:0:1:0:0:0:0:0:0:0:0 2 2.564103

19 1:0:1:1:1:1:0:0:0:1:1:1:0 2 2.564103

2 0:0:0:0:0:0:0:0:0:1:1:1:0 2 2.564103

24 1:1:0:0:1:0:0:0:0:1:1:1:0 1 1.282051

22 1:1:0:0:0:0:0:0:0:1:1:1:1 1 1.282051

21 1:1:0:0:0:0:0:0:0:0:0:0:0 1 1.282051

20 1:0:1:1:1:1:0:0:0:1:1:1:1 1 1.282051

18 1:0:1:1:1:1:0:0:0:0:0:0:0 1 1.282051

13 1:0:0:0:0:0:0:0:1:0:0:0:0 1 1.282051

12 1:0:0:0:0:0:0:0:0:1:1:1:1 1 1.282051

10 0:0:1:1:1:1:0:0:0:1:1:1:0 1 1.282051

9 0:0:0:0:1:1:0:0:1:0:0:0:0 1 1.282051

8 0:0:0:0:1:1:0:0:0:0:1:1:0 1 1.282051

6 0:0:0:0:1:0:1:1:1:0:0:0:0 1 1.282051

5 0:0:0:0:1:0:0:0:0:1:1:1:0 1 1.282051

3 0:0:0:0:0:0:0:0:0:1:1:1:1 1 1.282051
